# At the Deep End of dental inequality

**DOI:** 10.1038/s41415-025-8503-z

**Published:** 2025-08-08

**Authors:** Morgan J. Beeson, Christopher R. Vernazza, Sarah L. Sowden

**Affiliations:** 41415410951001 Newcastle University Business School, Newcastle University, UK; 41415410951002 School of Dental Sciences, Newcastle University, UK; 41415410951003 Population Health Sciences Institute, Newcastle University, UK

## Abstract

Due to the inverse care law, the crisis faced by NHS dentistry in England is felt most keenly by socioeconomically disadvantaged communities and the dental practices providing their care. Many challenges faced by these dental practices are shared by practitioners across the healthcare system. General medical practitioners (GMPs) in socioeconomically disadvantaged communities have set up local ‘Deep End' networks which advocate for patients and staff, share learning and best practice, and develop interventions for improving care and reducing practitioner burnout. Beginning in Glasgow, the Deep End movement is now global, spreading beyond GMPs to other areas of the healthcare system. The establishment of Deep End networks for general dental practitioners could serve as a powerful movement for identity, advocacy and action for socioeconomically disadvantaged communities and their dental practice teams. By adapting the GMP definition of the Deep End, the Deep End of dentistry in England is defined as the 10% of contracts treating the greatest proportion of deprived patients based on NHS England's inequalities framework, CORE20PLUS5. Administrative data from the NHS Business Services Authority is used to identify the Deep End. Advice for dental teams looking to set up a local Deep End dental network is given.

## Key points

- Groups of general medical practitioners in areas of widespread socioeconomic disadvantage have set up local ‘Deep End' networks to counter health inequality.
- Dental teams should consider establishing their own Deep End networks which could be a powerful movement for identity, advocacy and action for socioeconomically disadvantaged communities and the dental practice teams that serve them.
- This article provides interested dental team members with practical advice on how to set up a local Deep End network, including the publicly available data and methods required to identify potential members, important collaborators in policy and academia, and an approach to setting network objectives.

## Introduction

NHS (National Health Service) dentistry is in crisis, with reports of severe access problems for patients,^1^^,^^2^ practices struggling to recruit and retain staff,^3^^,^^4^ and charities required to step in.^5^^,^^6^ Due to the inverse care law, socioeconomically disadvantaged communities are hit hardest.^7^^,^^8^^,^^9^ Oral health and access to dentists are worse in socioeconomically disadvantaged communities.^10^^,^^11^ This leaves the people in these communities in more pain,^12^ with higher rates of spillover to secondary care, with more children having to undergo general anaesthetic^13^ and in some cases, people resorting to do-it-yourself dentistry.^14^ Additionally, the recent cost-of-living crisis is making oral hygiene products prohibitively expensive for some in the most socioeconomically disadvantaged communities, which will only worsen the problem.^15^ The experience of general dental practitioners (GDPs) operating in socioeconomically disadvantaged communities includes: treating many high-needs patients that the system does not adequately compensate;^9^ higher rates of non-attendance;^16^ and cultural and communication barriers,^17^ all coupled with the failure of national policy to respond adequately given the size of the problem.^18^ These are challenges that GDPs in more affluent areas do not face as regularly.

General medical practitioners (GMPs) working in socioeconomically disadvantaged communities report similar challenges, treating ‘left behind' communities in the face of workforce issues and demand that outstrips resources.^19^ In response, GMPs operating in communities where socioeconomic disadvantage is widespread have set up local ‘Deep End' networks to counter the inverse care law.^20^^,^^21^ The success of these networks (now a global movement) has not gone unnoticed by other areas of the healthcare system, with interest from emergency medicine and pharmacy.^22^^,^^23^ GDPs in socioeconomically disadvantaged communities should consider doing the same to benefit their practice and patients, now and in the ongoing conversations about the future direction of NHS dentistry.^24^^,^^25^

This article describes the Deep End movement's beginnings, purpose and successes. A methodology is then proposed for defining the Deep End of dentistry. The proposed method fits within the current GDP contractual arrangement and aligns with NHS England's current inequalities framework, CORE20PLUS5,^26^^,^^27^ of which oral health is a key target area of inequality for children and is central to the Change NHS consultation.^25^ The next steps required to set up a network are then discussed.

## The Deep End movement

Julian Tudor Hart's inverse care law states that ‘the availability of good medical care tends to vary inversely with the need for it in the population served. This inverse care law operates more completely where medical care is most exposed to market forces, and less so where such exposure is reduced'.^28^ This means that, without intervention, healthcare resources will concentrate away from socially disadvantaged communities, despite them needing more.

**Fig. 1:**
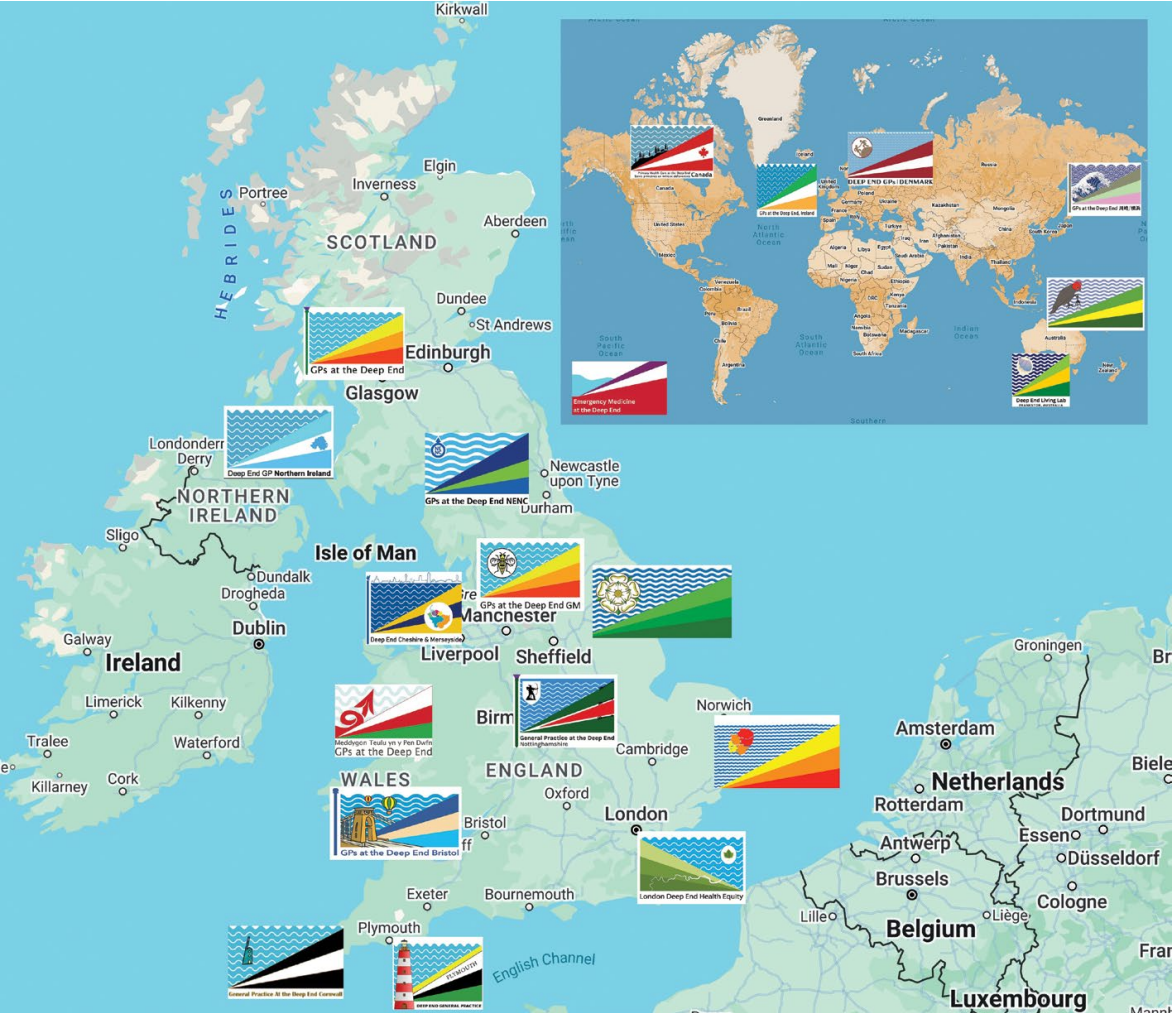
Map of UK and international Deep End networks marked by their network logos which share a common theme -depicting life at the Deep End as struggling to keep your head above water at the bottom end of the socioeconomic gradient. To date, GMP networks have been set up within the UK in: Scotland; NENC; Northern Ireland; Yorkshire and Humber; Greater Manchester; Cheshire and Merseyside; Nottinghamshire; Wales; East of England; London; Bristol; Plymouth; and Cornwall. Internationally, GMP networks have been set up in the Republic of Ireland, Denmark, Australia, Canada and Japan. A network has also been set up for emergency medicine. The networks and their logos are well-documented in the Deep End International Bulletin. Network logos used with permission.^33^^,^^46^ Map data ©2025 GeoBasis-DE/BKG (©2009), Google

Inspired by Tudor Hart, GMPs, initially in Glasgow, started regional Deep End networks over a decade ago to recognise and disrupt the inverse care law in their own communities.^29^ Deep End networks are GMP-led groups, bringing together practice nurses, managers and admin staff serving areas of widespread socioeconomic disadvantage (termed ‘blanket deprivation') to tackle health inequality. Noticing the success in Glasgow, other networks have formed. In the North East and North Cumbria (NENC) region, a number of GMP champions, alongside those working in public health, came together to form a network in partnership across the NHS, local government and academia. As Figure 1 shows, the movement has grown, with 11 networks across the United Kingdom (UK), and it is now an international movement, with nine countries represented, including Canada, Australia and Japan. The networks meet regularly to provide updates and share successes and learning which are reported in an international bulletin series.^30^^,^^31^

Interviews with GMPs and allied health professionals from the NENC network highlighted that life at the ‘Deep End' is characterised by treating ‘left behind' communities that have low expectations for their health and services they could receive which takes place in the presence of workforce issues and a demand/resource mismatch.^19^

In the face of that, there is an aspiration to do things differently.^19^ The purpose of these networks is to advocate for patients and staff, to share learning and best practice, to research and develop practical interventions for improving patient care, and to reduce the risk of practitioner burnout.^32^ Many networks have internally or externally funded fellowships for their staff to develop and enact interventions specific to their local needs.^33^ Efforts often revolve around four areas: workforce, education, advocacy and research (WEAR).^34^ For example, after the inception of the NENC Deep End network, co-design research with GMPs and allied health professionals was used to identify the network's initial key priority areas of focus, including recruitment,^35^^,^^36^ opioid and gabapentinoid deprescribing,^37^ and mental health.^38^ Pilot programmes were co-designed, enacted and evaluated for each key priority area, with the findings shared to inform activity in other practices within the network and beyond. The Deep End networks have begun working with medical schools to engage future doctors and offering placements.^33^ The lack of research participants coming from socioeconomically disadvantaged areas is a barrier to reducing health inequality.^39^ In response, increasing involvement and engagement of those living in Deep End communities in shaping research, interventions and producing guidance into reducing health inequality has also been central to Deep End general practice network activity.^40^^,^^41^

The problems described by GMPs are likely familiar to GDPs in those very same communities. The solutions GMP networks are pursuing are well-aligned with the proposals of existing inclusion oral health frameworks which promote the adjustment of service delivery to the community's needs, research and education.^42^ Setting up Deep End networks for GDPs could be part of the solution by complimenting existing inequality focused services,^43^ and by advocating for fair resources in the next iteration of GDP financing. As these communities have the lowest ability to pay for private dental care, it is important to preserve the NHS dental practices that provide their care in the face of potential dental deserts.

## Where is the Deep End of dentistry?

The method we propose here for defining which GDPs are classified as being within the ‘Deep End' of dentistry is adapted from methodology used to define some of the Deep End general medical practice networks in England. There are five steps in our process:

1. NHS dentistry in England is split into contracts
2. The patient list for each contract is defined as the number of unique patients receiving at least one course of treatment in the previous three years
3. A patient is categorised as CORE20 (deprived) if they live in one of the 20% most deprived of the 32,844 Lower Super Output Areas (LSOAs; a kind of census zone) based on standard deprivation scores (Index of Multiple Deprivation [IMD] 2019 rankings)^44^
4. All contracts nationally are ranked by the proportion of the patient list for each contract that are CORE20 patients
5. The Deep End is the 10% of contracts with the greatest proportion of CORE20 patients.

For the GMP definition, patients are characterised as deprived if they live in one of the 15% most deprived LSOAs. The choice of 20% most deprived for GDPs was made to align with NHS England's inequality programme, which focuses on the 20% most deprived LSOAs in England, or the ‘Core 20'.^26^

The NHS Business Services Authority (BSA) provided, through a freedom of information request (FOI-01065, Dental Services, NHSBSA, Copyright 2022), data on contracts in England, including commissioner name, primary treatment address and the count of unique patients seen between 1 April 2017 and 31 March 2020 by IMD vigintile (5% bands). A unique patient is defined by the patient's name and date of birth. While a single patient would be counted twice or more by attending multiple practices in the time period, the impact is assumed to be small. The period was chosen to avoid the impact of COVID-19 and would need to be updated over time. The IMD vigintile is based on the 2019 IMD score of the LSOA of the patient's home address postcode. Patients without a postcode recorded, and therefore with no IMD score, remain in the total count of patients when calculating the proportion of CORE20 patients. To maintain anonymity, patient counts less than or equal to five are censored by the BSA and recoded as zero. The data consists of 8,907 contracts, with a positive number of patients seen. The data were merged with contract information for the 2015-2022 financial years from the BSA website to identify and remove contracts that do not provide units of dental activity, Community Dental Services (CDS), or those providing care in prisons, hospitals and universities. These services are identified by the contract name, including key words such as ‘HMP', ‘hospital', ‘CDS', etc. This leaves a total of 8,384 contracts.

**Fig. 2:**
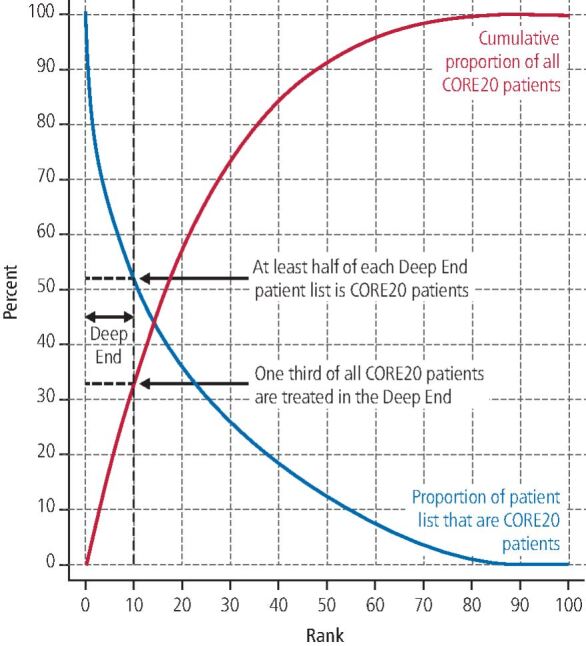
Proportion of patient list that are CORE20 patients (blue) and cumulative proportion of all CORE20 patients (red) by rank of proportion of patient list that is CORE20 from highest to lowest

By following the methodology above, we identified 838 contracts as the Deep End of dentistry. Figure 2 depicts Steps 4 and 5 by presenting all contracts ranked along the x-axis from the highest to lowest proportion of CORE20 patients. The left-most 10% is the Deep End marked by the vertical dashed line. The proportion of CORE20 patients (blue line) treated under Deep End contracts ranges from 100% down to 52%, with a mean of 68%. The cumulative proportion of all CORE20 patients (red line) treated under Deep End contracts is one-third. The difference with contracts not in the Deep End is stark. The mean proportion of CORE20 patients treated is just 14% for the remaining 90% of contracts that are not in the Deep End. The last 10% of ranked contracts treat no CORE20 patients (i.e., only those from areas of relative affluence).

**Fig. 3:**
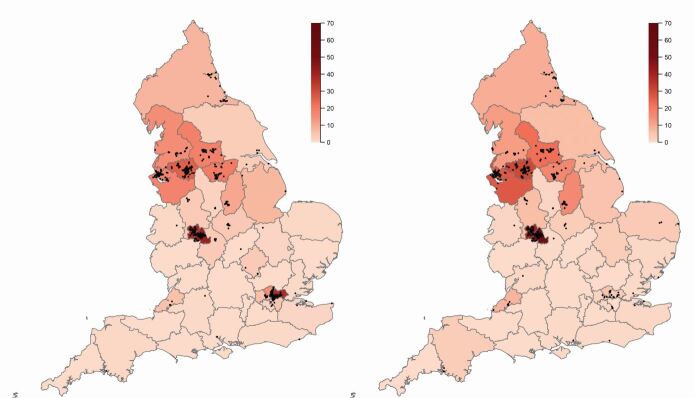
Map of the Deep End for GDPs (left) and GMPs (right). Black points mark contract/practice locations and shading shows the percentage of contracts/practices in each ICB that are Deep End. Source: Office for National Statistics licenced under the Open Government Licence v.3.0. Contains OS data ©Crown copyright and database right 2024

The location of the Deep End contracts (red points) are mapped on Figure 3 where primary treatment addresses are available. The shaded colours represent the percentage of contracts in each integrated care board (ICB) that are in the Deep End. To give a comparison, Figure 3 also maps the Deep End for GMPs when the related methodology based on the same 20% deprivation threshold is applied. The distribution of Deep End practices is similar for GDPs and GMPs which are clustered around Liverpool, Manchester, Leeds, Sheffield, Birmingham, London, and the coast between Newcastle-Upon-Tyne and Middlesborough. There are far more Deep End GDPs in London than Deep End GMPs.

## Next steps

For GDPs interested in setting up a Deep End network, Butler *et al.,*^32^ through a scoping review, outlines seven themes that arise when setting up a Deep End network for GMPs:

1. Quantify where the Deep End is
2. Host a meeting where participants establish the group's future objectives
3. Secure funding; desirable but not essential
4. Establish a smaller steering group
5. Collaborate with academic general practice
6. Decide on membership eligibility
7. Adopt a Deep End logo.

This article has focused on Theme 1 at a national level. Themes 2-7 are driven by the member practices themselves. Networks are usually local and therefore a geographical boundary is needed to be set. GMP networks range in size, from city to country level; although, the county or region level is most common in England. NHS ICB regions are a good candidate for setting geographical boundaries and as potential sources of funding (Theme 3) as they are responsible for local commissioning, financing and tackling inequalities. The rules to define the Deep End described above are not hard and fast, and border cases are usually considered. Other aspects of geographic inequality, such as living in rural and coastal areas, may be considered.

The common objective between networks is advocacy for patients and practitioners. Theme 2, however, points out that each network defines its own objectives and membership through co-design so that the focus is on local issues with a smaller steering group (Theme 4) to keep the network accountable to the objectives. Theme 5 is an important relationship for any Deep End network which can facilitate research and develop practical interventions to improve patient care. As an example, the NENC Deep End primary care network involved researchers from the outset to gather stakeholder opinions to set the initial objectives of the network^19^ and have an ongoing co-design and evaluation research-practice partnership.^45^ Under Theme 6, some networks are limited to general practitioners but others include practice managers, nurses, public health colleagues, educators and students. Finally, Theme 7 describes the network flags, which, as Figure 1 shows,^46^ provide a unique identity to each network but also serve to unify and link the networks together through the common theme of a gradient under water which represents the struggle to keep your head above water at the ‘deep end'.

An initial meeting for a Deep End Dental group in the NENC has been held to progress to Theme 2 for dentistry in one local area.

## Conclusion

If NHS dentistry in England is to ‘build back fairer',^24^ the establishment of Deep End networks for GDPs, as has been the case for GMPs, could serve as a powerful movement for identity, advocacy and action for socioeconomically disadvantaged communities and the dental practice teams that serve them. This article provides a definition of the Deep End of dentistry, sets out a methodology for identifying Deep End dentistry contracts across England, and shows the distribution of these nationally.
